# Is an Immunosuppressive Microenvironment a Characteristic of Both Intra- and Extraparenchymal Central Nervous Tumors?

**DOI:** 10.3390/pathophysiology28010004

**Published:** 2021-01-08

**Authors:** Amina Soltani, Bela Kajtar, El Husseiny Mohamed Mahmud Abdelwahab, Anita Steib, Zsolt Horvath, Laszlo Mangel, Luca Jaromi, Judit E. Pongracz

**Affiliations:** 1 Department of Pharmaceutical Biotechnology, Faculty of Pharmacy, University of Pecs, 7624 Pecs, Hungary; aminasoltani8@gmail.com (A.S.); elhusseiny.mohamed@pte.hu (E.H.M.M.A.); jaromi.luca@pte.hu (L.J.); 2 Szentagothai Research Centre, University of Pecs, 7624 Pecs, Hungary; 3 Medical School and Clinical Centre, Department of Pathology, University of Pecs, 7624 Pecs, Hungary; kajtar.bela@pte.hu; 4 Humeltis Ltd., 7624 Pecs, Hungary; anita.steib@humeltis.com; 5 Medical School and Clinical Centre, Departments of Neurosurgery, University of Pecs, 7623 Pecs, Hungary; horvath.zsolt@pte.hu; 6 Oncotherapy, Medical School and Clinical Centre, University of Pecs, 7624 Pecs, Hungary; mangel.laszlo@pte.hu

**Keywords:** meningioma, glioblastoma, immune microenvironment, immune suppression

## Abstract

In spite of intensive research, the survival rates of patients diagnosed with tumors of the central nervous system (CNS) have not improved significantly in the last decade. Immunotherapy as novel and efficacious treatment option in several other malignancies has failed in neuro-oncology likely due to the immunosuppressive property of the brain tissues. Glioblastoma (GBM) is the most aggressive malignant CNS neoplasm, while meningioma (MNG) is a mainly low grade or benign brain tumor originating from the non-glial tissues of the CNS. The aim of the current preliminary study is to compare the immune microenvironment of MNG and GBM as potential target in immunotherapy. Interestingly, the immune microenvironment of MNG and GBM have proved to be similar. In both tumors types the immune suppressive elements including regulatory T cells (Treg), tumor-associated macrophages (TAM) were highly elevated. The cytokine environment supporting Treg differentiation and the presence of indoleamine 2,3-dioxygenase 1 (IDO1) have also increased the immunosuppressive microenvironment. The results of the present study show an immune suppressive microenvironment in both brain tumor types. In a follow-up study with a larger patient cohort can provide detailed background information on the immune status of individual patients and aid selection of the best immune checkpoint inhibitor or other immune modulatory therapy. Immune modulatory treatments in combination with IDO1 inhibitors might even become alternative therapy for relapsed, multiple and/or malignant MNG or chemo-resistant GBM.

## 1. Introduction

In 2019 about 87,000 people were diagnosed with primary brain tumors in the United States alone. An estimated 26,000 cases were malignant and 61,000 cases were so called benign [1]. In the current study, we focused on the immune microenvironments of two main types of brain tumors with unrelated histology and origin, namely glioma and meningioma. We compared their immune microenvironments to evaluate the potential use of currently available immune therapies.

Histologically the two tumor types that were selected for the study couldn’t be more different. The malignant gliomas originated from glia cells (astrocytic, ependymal and oligodendrocytic types) and are categorized as low-grade gliomas (LGG grades I and II) and high-grade gliomas (HGG grades III and IV) [2,3]. Glioblastomas (GBM), the most frequent parenchymal tumors are grade IV gliomas that are the most aggressive form of the disease [4]. The relatively slow growing and relatively benign meningiomas (MNG), the most frequent amongst the extra-neural tumors spring from the extra-parenchymal part of the CNS, from the three layers of membranes (meninges) that cover the brain and the spinal cord. These tumors are generally slow-growing (70–80% are grade I) but can become atypical (5–20% grade II) or even malignant (1–3% grade III) [5].

Despite all the efforts to find effective therapy, currently the median survival of GBM is 15 months [6]. Surgery is typically the first therapeutic approach followed by radiotherapy and/or chemotherapy which is almost invariably involves temozolomide (TMZ) treatment [4,7,8,9]. TMZ together with radiotherapy has improved the 2-year survival rate of GBM to 18% compared to 4% of radiotherapy alone [10]. Interestingly, there is less therapeutic benefit using TMZ for MNG [11] due to an active DNA repair mechanism predominantly present in MNG [12]. As a result, surgery and radiotherapy are used primarily to treat the disease. Surgery, however, is not always possible due to the eloquent location or irresectability of the tumor, which explain the increased role of different types of radiotherapy as the most frequently applied alternative or additive local treatments [13,14].

Not surprisingly, in the latest years great expectations were looked forward to immune checkpoint blockers [15,16] or immune cell activators [17] as a potentially more effective therapeutic route for treating brain tumors. Immune checkpoint inhibitors reformed the treatment of many types of cancers including non-small cell lung cancer, melanoma, renal and colorectal cancer [18] by inhibiting suppression of cytotoxic T cell activity via blocking PD-1 and PDL-1 or B7 and CTLA-4 interactions [19]. There are also great expectations surrounding the targeted activation of natural killer (NK) cells in tumor treatment [20].

Although actively investigated [21], there is still limited knowledge about targetable immune processes of CNS tumors. Certainly, the presence of neutrophils, T and B lymphocytes, NK cells and myeloid-derived suppressor cells (MDSC) have been reported in brain tumors in recent research articles [22,23,24]. Several recent reviews have dealt with the special immune microenvironment of the CNS and a need for active research to overcome the special location of CNS tumors. A fundamental marked partition can be differentiated between the brain parenchyma and meningeal spaces. The brain parenchyma is guarded by perivascular macrophages and microglia, the tissue-resident macrophages of the brain, while the meningeal spaces have a more diverse immune repertoire [25].

Additional challenge in treating CNS tumors is the existence of the blood brain barrier (BBB) phenomenon [26]. To date promising immunotherapy modalities that have passed clinical trials for other neoplasms have not been proved successful at overcoming the BBB protective effect in treating CNS tumors [27]. Although extensive research is ongoing to find suitable solutions including the use of nano-immuno-conjugates that can carry checkpoint inhibitors across the BBB with the aim to switch off the immune suppressive macrophages and regulatory T cells. However, the use of such applications have not reached clinical trials yet [28]. Despite all the efforts GBM remains a largely unmet medical need, while the effective therapy for MNG continues to be surgery. Both GBM and MNG could benefit from immunotherapy if the microenvironment is characterized in detail.

In the present preliminary study, we aimed to compare the immune microenvironment of primary MNG and GBM to distinguish the immune status of the two CNS tumors and to provide a better insight of their immune microenvironment that could potentially be targeted in clinical applications. First, characteristic immune markers in both MNG and GBM were compared to the normal brain, then deviation from normal marker expressions were correlated with markers in MNG and GBM. In the attempt to investigate whether similar immune modulators could be used in both tumor types, we designed a “bulk cell approach” exploratory study to characterize the overall immune microenvironment laying the foundation for a more detailed investigation in the future, which will allow to decide whether the same or similar immunotherapy approach could be used in both MNG and GBM.

## 2. Materials and Methods

### 2.1. Ethical Statement

Brain tumor samples were collected at the Departments of Neurosurgery and Pathology, Clinical Centre, University of Pecs, Hungary. In accordance with the Declaration of Helsinki patients had given written informed consent and the project was approved by the Medical Research Council, Hungary (0194/16 (10833-/2016/EKU).

### 2.2. Patient Samples

Individual patient data are summarized in Table 1.

### 2.3. RNA Isolation and Reverse Transcription

Total RNA was isolated from frozen grade IV human GBM and MNG samples using NucleoSpin RNA isolation kit (Macherey-Nagel, Düren, Germany). RNA concentration was measured by Nanodrop 2000 (ThermoFisher Scientific, Waltham, MA, USA). Reverse transcription was performed using random primers and a high capacity RNA to cDNA kit (ThermoFisher Scientific, Waltham, MA, USA). All generated cDNA samples were stored at −20 °C until used. Total RNA of five pooled normal human brain samples was purchased from a commercial source (BioChain Institute, San Francisco, CA, USA). All generated cDNA samples were stored at −20 °C until used.

### 2.4. Real-Time Quantitative Polymerase Chain Reaction (qRT-PCR)

qRT-PCR reactions were carried using Luminaris Color HiGreen qPCR master mix (ThermoFisher Scientific) and amplification was made by PikoREAL 96 PCR system (ThermoFisher Scientific). The reference genes were β-actin and glyceraldehyde 3-phosphate dehydrogenase (GAPDH) by taking the average of their Ct values. The relative quantification (RQ) was calculated compared to gene expression levels of the normal human brain. The primer sequences are summarized in Table 2.

### 2.5. Hematoxylin-Eosin Staining

Five µm thick tissue sections were stained in Mayer’s hematoxylin solution (Sigma-Aldrich, St. Louis, MO, USA) for 10 min, washed, then exposed to 0.25% acetic acid and eosin solution. Sections were mounted using Vectashield mounting medium (Vector Laboratories, Burlingame, CA, USA). Images were taken using an Eclipse Ti-U inverted microscope (Nikon Inc., Tokyo, Japan).

### 2.6. Immunohistochemistry

Five µm thick slides were cut from formalin-fixed, paraffin embedded tissue blocks corresponding to the surgical samples used for qRT-PCR. First, the slides were rinsed in heated xylene and were washed with a descending series of ethanol (97%–80%–70%–50%) to remove paraffin. After deparaffinization the slides were rehydrated by distilled water and DAKO antigen Target Retrieval Solution (DAKO, Agilent, Santa Clara, CA, USA) at 97 °C for 20–30 min and endogenous peroxidase activity was blocked for 15 min with Tris Buffer Saline (TBS, pH 7.4) containing 3% H_2_O_2_. Slides were washed three times with containing TBS Tween (0.05%, pH 7.4). Pre-blocking was carried out with 3% bovine serum albumin (BSA) in TBS for 20 min before overnight incubation with the appropriate primary antibody at 4 °C. Slides were then washed with TBS for three times. The reactions were visualized using Envysion System (DAKO). For nuclear counterstaining, hematoxylin staining was performed. Finally, slides were mounted with Faramount Aqueous Mounting Medium (DAKO). Histological evaluation was performed with the help of Panoramic MIDI digital slide scanner (3DHistech, Budapest, Hungary). The number of positive cells was assessed per mm^2^ except for the CD68 positive cell count that was assessed per 0.08 mm^2^. Image analysis was performed using the ImageJ software with the IHC toolbox plug-in. The list of antibodies and dilutions are summarized in Table 3.

### 2.7. Immunofluorescent Staining

5 µm thick slides were cut from formalin-fixed, paraffin embedded tissue blocks corresponding to the surgical samples used for qRT-PCR. After deparaffinization and antigen retrieval the sections were pre-blocked with 5% BSA in TBST for one hour before applying primary antibodies anti-CD19 and anti-CD45 for overnight at 4 °C. CD19 and CD45 were detected using an anti-mouse Alexa 488 (1:200) and anti-rabbit Alexa 555 (1:200) (ThermoFisher Scientific), respectively. Nuclei were counterstained with dapiprazole hydrochloride (DAPI) (ab142859) (1:1000) (Abcam Plc., Cambridge, UK). Images were obtain using an Olympus IX-81 (OLYMPUS Corporation, Tokyo, Japan) fluorescence microscope. The list of antibodies and dilutions are summarized in Table 3.

### 2.8. Statistical Analysis

Statistical analysis was performed with SPSS version 20 software and figures were generated using GraphPad Prism 8 (2018, GraphPad Software Inc., La Jolla, CA, USA). Data are presented as 1/dCt individually and average ± standard error of mean (SEM) using one-way and two-way ANOVA. *p* < 0.05 was considered as significant.

## 3. Results

### 3.1. Variable Infiltration of T, B, NK Cells and Macrophages into MNG and GBM

**Figure 1:**
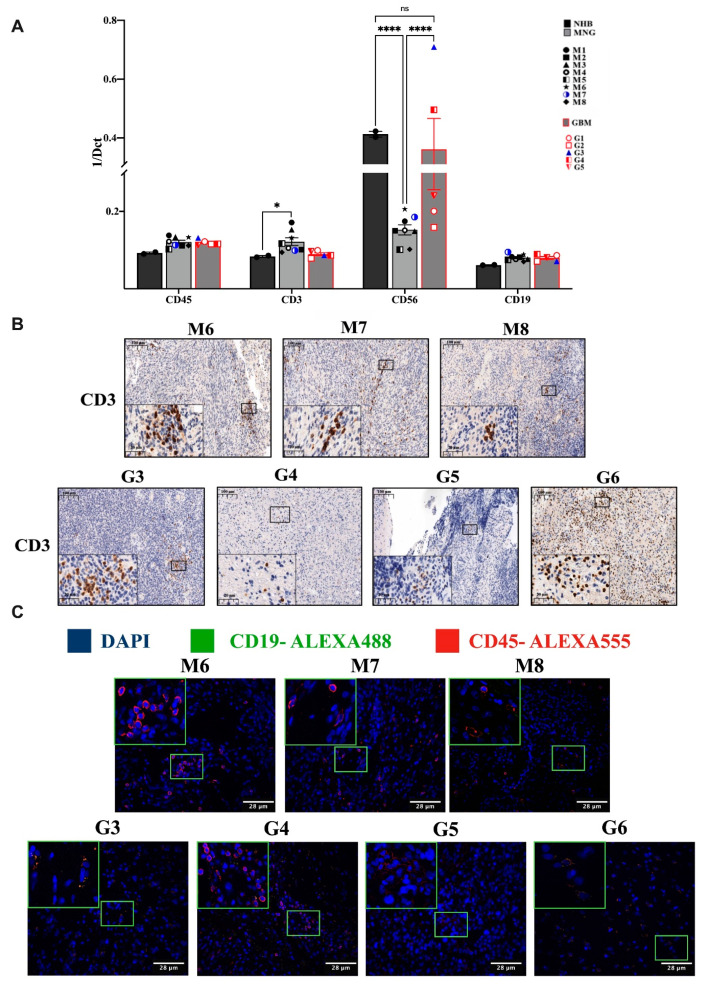
Infiltration of immune cell populations into MNG and GBM. (**A**) mRNA levels of the general leukocyte antigen CD45 and general immune cell subpopulation CD3^+^ T, CD56^+^ NK and CD19^+^ B cell markers in MNG (*n* = 8) and GBM (*n* = 5). Data are presented as 1/dCt individually and as average ± SEM. Significant changes are marked as * and **** (*p* < 0.05 and *p* < 0.0001, respectively). (**B**) Immunohistochemistry staining of CD3 T-cell population in both brain tumor types (GBM and MNG), magnification ×20 and ×40, size bar 100 and 20 μm respectively. (**C**) Immunofluorescence staining of the general leucocytes marker CD45 and of CD19 in MNG (*n* = 3) and GBM (*n* = 4), magnification ×40, size bar 28 μm. Only red staining can be detected in MNG and yellow (overlapping red and green) in some GBM samples.

qRT-PCR analysis of both MNG and GBM samples have shown a slight increase of the CD45^+^ white blood cell infiltration marker (Figure 1A). To identify the main cell types within the white blood cell population, expression of CD3^+^ T, CD56^+^ NK and CD19^+^ B cell markers were screened (Figure 1A). The transcript levels of the T cell marker CD3 in MNG were significantly higher than in the normal brain control, while in the GBM samples CD3 expression was not different from the normal brain control (Figure 1A). The NK cell marker CD56 is significantly reduced in all MNG samples compared two both normal brain and GBM (Figure 1B). The presence of mRNA level of CD19 B cell marker was observed at equally low levels both in MNG and GBM samples highly similar to normal brain samples (Figure 1A). Immunohistochemistry supported the initial findings, as the tested individual MNG samples had generally higher T cell marker CD3 at protein levels than what was detected in GBM (Figure 1B). Neither the tumors, nor the infiltrating CD45^+^ lymphocytes stained positive for CD19. Certain areas of GBM sections have shown some congregation of CD19^+^CD45^+^ double positive cells, while such areas were not found in MNG (Figure 1C). The lack of B cells in MNG and GBM was also supported by negative staining for CD79a and the expression of PAX5 (Supplementary Figure S1).

### 3.2. The Immune Microenvironment Is Actively Suppressive in Both MNG and GBM

**Figure 2:**
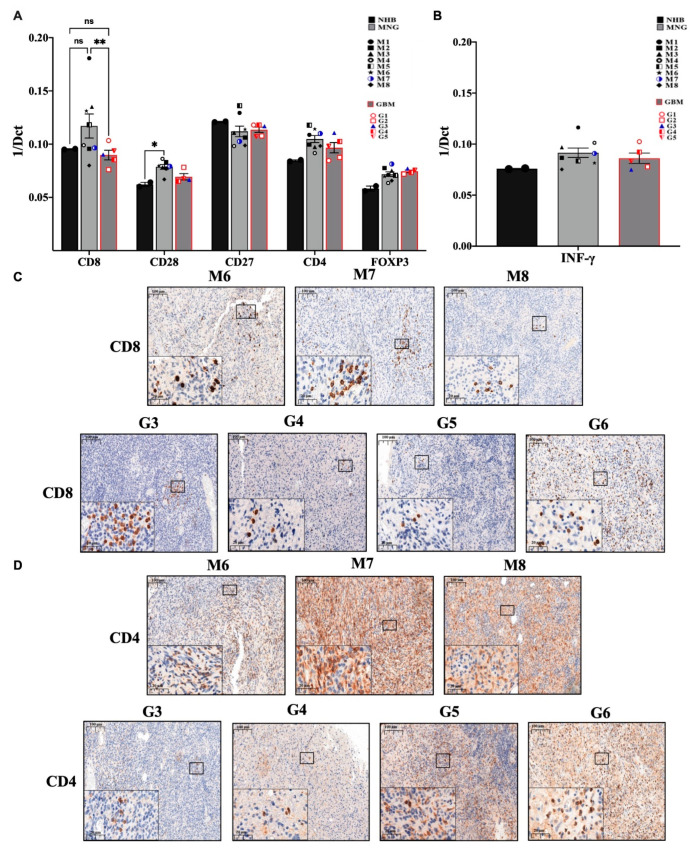
Evaluation of the immune profile of MNG and GBM. (**A**) mRNA expression levels of CD8 cytotoxic T-cells and CD4 T-helper cells, CD28 as costimulatory signal transducer for T-cells survival and activation, as well as FOXP3 marker characteristic for regulatory T cells. (**B**) The anti-tumor interferon-gamma (INF-γ) mRNA expression levels were evaluated in both brain tumor microenvironments. Data are presented 1/dCt individually and as average ± SEM. Significant changes are marked as * and ** (*p* < 0.05 and *p* < 0.001, respectively). (**C**) Immunohistochemistry staining of CD8 was performed in MNG (*n* = 3) and GBM (*n* = 4) samples, magnification ×20 and ×40, size bar 100 and 20 μm respectively. (**D**) Immunohistochemistry staining of CD4 cells in MNG and GBM, magnification ×20 and ×40, size bar 100 and 20 μm respectively.

In the initial screening, the immune microenvironment appeared different at the level of T and NK cells in MNG and GBM. At first glance these findings might explain some of the characteristically different behavior of the tumors, indicating a more active tumor suppressive microenvironment in MNG. The cytotoxic T cell marker (CD8) is markedly increased in MNG compared to normal brain control and significantly higher than in GBM samples (Figure 2A). CD28, a co-stimulatory molecule essential for T cell activation is also present in both tumor types but only significantly increased in MNG compared to normal brain control (Figure 2A). The pro-inflammatory and anti-tumor interferon gamma (INF-γ) mRNA levels were also slightly increased (Figure 2B) in both MNG and GBM samples compared to normal controls. Although CD8^+^T cells were only found in certain areas of the tumors (Figure 2C), a potentially successful immune checkpoint intervention appeared to be a distinct possibility for MNG.

Further analysis of T cell markers revealed, that the CD4^+^ helper T cell marker message levels were slightly increased in both tumor types along with the regulatory T cell marker FOXP3 (Figure 2A). The presence of CD4^+^ T cells were also supported by immunohistochemistry (Figure 2D). In contrast to the CD8^+^ T cells marker that localized to specific tumor areas, evenly distributed CD4 staining was detected (Figure 2C,D) in both MNG and GBM samples indicating the presence of CD4^+^ helper T cells throughout both tumor tissues that are more likely to be immune suppressive in nature (regulatory T cells (Treg)) due to the elevated levels of FOXP3.

**Figure 3:**
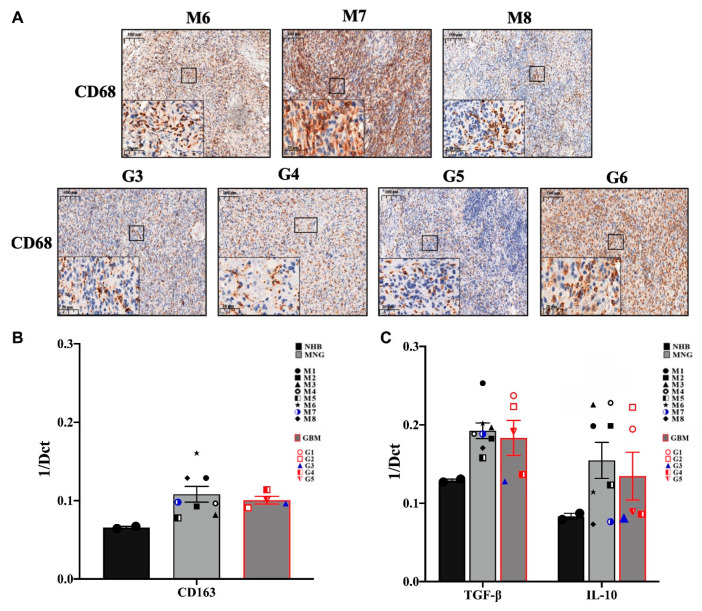
Tumor-associated macrophages (TAM) and immunosuppressive cytokine expressions in MNG and in GBM. (**A**) Immunohistochemistry staining of CD68 indicate the higher protein expression of TAM in both tumors, magnification ×20 and ×40, size bar 100 and 20 μm respectively. (**B**) CD163 m-RNA expression levels which represents TAM marker in MNG and GBM. (**C**) mRNA expression levels of anti-inflammatory cytokines IL10 and transforming growth factor β (TGFβ) in MNG and GBM samples. Data are presented as 1/dCt individually and as average ± SEM. Significant changes are marked as * (*p* < 0.05).

Immunohistochemistry of the tumor-associated macrophage (TAMs) marker CD68 has started to reveal less hope for successful application of immunotherapy (Figure 3A). Both tumor types, were strongly and evenly positive for CD68 [29]. qRT-PCR analysis of another TAM marker CD163 strongly supported the initial observation (Figure 3B), as both tumor types expressed CD163 message levels way above normal controls. One of the major functions of TAMs is suppressing the T-cell mediated anti-tumor immune response via expression of IL-10 and transforming growth factor β (TGFβ) [30]. The anti-inflammatory TGFβ and IL10 were expressed at higher levels in both MNG and GBM tumors than in normal brain (Figure 3C) indicating the active presence of TAMs. As both cytokines are involved in creating the immune-suppressive environment by inhibiting the polarization of naïve T cells into Th1 and NK cells, the low level of NK cell marker CD56 in MNG was supported by the increased message levels of the above cytokines (Figure 1B). Additionally, IL10 is known to be over-expressed not only by CD163^+^ TAMs, but also by immunosuppressive regulatory T cells (CD4^+^ FOXP3^+^Treg). As the Treg marker FOXP3 message levels were higher in both tumor types than in normal controls, the results have indicated an actively immunosuppressive microenvironment in both MNG and GBM. Additionally, although in MNG cytotoxic T cell levels were higher than in GBMs, and expression of the co-stimulatory CD28 was also present in both tumor types, the mRNA levels of CD27, a member of the tumor necrosis factor (TNF) receptor superfamily and co-stimulatory immune checkpoint molecule for activated T cell survival was highly presented compared to CD28 (Figure 2B). As CD27/CD70 interaction promotes lymphocytes apoptosis, it is likely that activated immunosuppressive lymphocytes persist in both MNG and GBM. CD27 also aids differentiation of plasma cells from B cells if CD27 can interact with its ligand CD70.

As TAMs can directly suppress T cell function by the induction of programmed death-ligand 1 (PD-L1) [31] and B7-homolog expression [32] the expression of immune checkpoint therapy targets were tested. Interaction of PDL-1 with programmed cell death protein 1 (PD-1) and B7 with cytotoxic T-lymphocyte antigen 4 (CTLA-4) can block T cell activity, respectively, and lead to suppression of cytotoxic T cell activation.

### 3.3. Immune Checkpoint Targets in MNG and GBM

**Figure 4:**
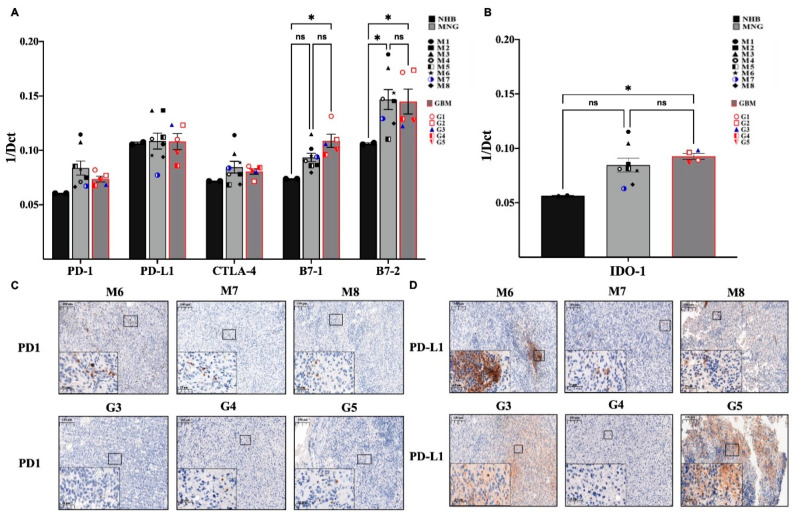
Immune checkpoint molecules that mediate immune therapy response in MNG and GBM. (**A**) mRNA expression levels of PD-1, PD-L1, CTLA-4, B7-1, and B7-2 in MNG and GBM patient samples. (**B**) mRNA expression levels of Indoleamine 2,3-dioxygenase-1 (IDO-1) were tested in MNG and GBM. Data are presented as 1/dCt individually and as average ± SEM. Significant changes are marked as * (*p* < 0.05). (**C**,**D**) Both MNG and GBM samples were stained for PD-1 and PD-L1, magnification ×20 and ×40, size bar 100 and 20 μm respectively.

The expression of immune checkpoint targets (PDL1-PD1, B7-CTLA4) [31,32] in MNG and GBM are different. qRT-PCR analysis revealed great variability of T-cell receptor PD1 expression in individual MNG cases (Figure 4A) were somewhat higher than PD1 expression in GBM (Figure 4A). Overall, neither MNG nor GBM stained strongly for PD1 protein, although there were more PD1 positive T cells in MNG than in GBM (Figure 4C). As PD1 is found on T cells, it also shows that higher CD8 levels in MNG might explain the difference in malignancy of the tumors. In contrast, while PDL1 message levels were similar to PD1 at mRNA, PDL1 was strongly stained at protein levels in both tumor types (Figure 4A,D) (Table S1). The pattern of the staining, however, was interesting as it resembled the staining of T cell markers CD3 (Figure 1C), CD8 (Figure 2C) and CD4 (Figure 2D). Staining was evenly distributed in GBM and localized to specific areas of the tumor in MNG (Figure 4D). To measure the other molecular pair of the immune checkpoint therapy targets, mRNA expression levels B7-1 (CD80) and B7-2 (CD86) and CTLA-4 were also tested (Figure 4A). Both B7-1 and especially B7-2 molecules were significantly increased in MNG and GBM compared to normal control. As the inhibitory CTLA-4 mRNA levels were slightly increased in both MNG and GBM compared to normal brain control, the immune suppressive microenvironment was supported further by the above findings (Figure 4A). Additionally, IDO1, the L-tryptophan metabolizing enzyme, was strongly expressed in MNG and significantly higher in GBM using normal brain as a control (Figure 4B). The metabolic product that is generated by IDO1 enhances the activities of CD4^+^FOXP3^+^ Treg cells and myeloid-derived suppressor cells, as well as promote angiogenesis.

## 4. Discussion

Better understanding of the microenvironments of various types of CNS tumors is essential for more effective therapies. In the present preliminary study we used traditional methods to investigate to unrelated CNS tumors for the potential application of immunotherapy. Although the main similarity of the two tumor types is the closed off and hard to reach environment of the CNS, our investigation revealed overall similarities in the immune microenvironment. Although single-cell sequencing could have characterized individual clonal dynamics and provided a snapshot of tumor heterogeneity, the accumulated scientific knowledge using such techniques have still not translated into improved treatment modalities [33]. Hence the traditional approach was deemed to be more suitable to provide more information for further, translational research directions. Currently, the primarily available option to treat MNG or GBM is still surgery, and even if the tumor is operable, the procedure might lead to memory loss, speech, and mobility problems. If the tumor is inoperable or in adjuvant setting radio- and chemotherapy are the remaining treatment options having different side-effects. Chemotherapy practically invariably involves TMZ, but only about 50% of GBM patients respond to TMZ treatment [34]. Unfortunately, TMZ also has various adverse reactions. MNG are even less responsive to TMZ [11], therefore it is not surprising that other systemic treatment options are actively investigated.

Immune checkpoint antibodies were hoped to revolutionize CNS tumor therapy, but the clinical results remained controversial [35,36]. To understand the reasons, several studies have investigated the immune microenvironment of GBM [37,38], but there was less focus on extra-parenchymal CNS neoplasms, like MNG. Heterogeneity of tumor infiltrating immune cells has also become the center of attention in an attempt to predict therapy responses, although in CNS tumors the information is still limited [39].

In our preliminary study we started the investigation with targets of immune checkpoint monoclonal antibodies. PDL1 protein expression was detected in both MNG and GBM, which would have indicated potentially successful application of the PDL1-PD1 immune checkpoint therapy for effective tumor elimination. While the ligand PDL1 was detected in both tumor types, the T cell inhibitory receptor PD1 was barely present. Additionally, CTLA4 a member of the CD28 family of receptors, a strong inhibitor of T lymphocyte co-stimulation was although elevated in some individual patient samples, generally its expression was not significantly increased making it questionable whether therapeutic monoclonal antibodies would successfully affect tumor cell growth in GBM or MNG if sufficient level of cytotoxic T cells were present in the tumor tissues [18,19,40,41,42]. While T cells are present in both tumor types, CD8^+^ cytotoxic T cells are only in abundance in MNG providing the false impression that MNG could be targeted with immune checkpoint therapy. The initial observation, however, is misleading. While certain tumors exploit the PD1-PDL1 and CTLA4-B7 T cell suppression system to evade immune recognition by expressing high levels of PDL1 [43,44], our data indicates that immune therapy could only be used if PDL1 molecules would be targeted by specific antibodies. In such cases the Fc region of the anti-PDL1 antibody would be accessible for immune cells with Fcγ receptors, such as NK cells. The low level of NK cell marker CD56^+^ in MNG and in most GBM samples, explains the lack of efficacy of NK cell targeting therapy [45]. Additionally, even the presence of NK cells doesn’t ensure their activated state as tumor-infiltrating immune cells such as dendritic cells (DCs), suppressive or tolerogenic macrophages and regulatory T (Treg) cells, can interfere with NK cell activation either through secretion of immunosuppressive cytokines or by interfering with receptor expression [46,47]. For instance, TGF-β is recognized as a main inhibitory cytokine of NK cells which limits the number and anti-metastatic function of NK cells and is highly expressed in the studied tumors. The microenvironment in both GBM and MNG are highly immunosuppressive as CD68^+^ and CD163^+^ anti-inflammatory M2 type TAM-s infiltrate both GBM and MNG. TAM-s, which secrete anti-inflammatory and immune suppressive cytokines (e.g., TGFβ and IL10), enhance the expansion of immune suppressive CD4^+^ Treg cells, inhibit the functions of CD8^+^ cytotoxic T and NK cells and similarly to tumor cells also express IDO1 [48]. IDO1 is a heme-containing enzyme that catalyzes the first and rate-limiting step in the kynurenine pathway, which is the O_2_-dependent oxidation of L-tryptophan to N-formylkynurenine. INFγ that is highly expressed in both GBM-s and meningiomas stimulates tissue macrophages to produce a higher level of IDO1, which via alteration of cytokine levels inhibits the proliferation of effector T cells. The immune-suppressive role of IDO-1 was supported by studies using Trp metabolites that induced differentiation of regulatory T cells and increased apoptosis of effector T cells via inhibiting the mechanistic target of rapamycin complex 1 (m-TORC1) [49,50].

Currently, several IDO-1 inhibitors including epacadostat, navoximod and BMS-986205 [13] are under clinical evaluation and the results are promising using IDO1 inhibitors in combination with anti-PD1 drugs in preclinical models of GBM [14]. Another study performed in a GBM mouse model using anti-PD-1, anti-CTLA-4 and IDO1 inhibitor combination showed a dramatic improvement in slowing down disease progression [51].

## 5. Conclusions

Overall, activation or inhibition of the immune system depends on the balance between co-stimulatory and co-inhibitory pathways. In aggressive tumors the immune system is often suppressed which ensures the survival of the tumor cells. It appears that combination therapies are necessary to overcome the strongly immune suppressive brain tumor milieu. Using the appropriate immune checkpoint inhibitors in combination with IDO1 inhibitors might be an alternative treatment for both parenchymal and extra-parenchymal therapy resistant brain tumors.

Although further studies are essential, the differences between MNG and GBM are clear. MNG has no NK cells so even targeted therapy using tumor specific antibodies would not activate NK cell that carry FcγR as there aren’t any NK cells to recognize the antibody and eliminate the tumor cell. Even macrophages –the other cell type with FcγR- are likely to be of TAM-s. The CD8^+^ cytotoxic T cells are present in MNG and they also express PD1, but the tumor is negative for PDL1 therefore immune checkpoint inhibition would not have any effect. The presence of the large number of helper CD4^+^ T cells, CD68^+^ TAMs and IDO1 point to immunosuppression. Although GBM have normal level of NK marker, GBM has no higher cytotoxic T cell level than normal and no PD1 staining. Meanwhile, there is an abundance of CD4^+^ helper T cells, CD68^+^ TAMs and significantly increased IDO1. Additionally, both MNG and GBM have significantly increased B7-2 (CD86) expression that is a ligand of CTLA-4 on T cells. CTLA-4 has the role to turn down T cell activation. As CTLA-4 is there on all T cells but its level is not higher than in the control in either MNG or GBM, it can also lead to a complex immunosuppressive signal. The immunosuppressive CD4^+^ Treg cells that are in abundance in both MNG and GBM do not express CTLA-4, it can lead to continuous activation of the immunosuppressive CD4^+^ Treg cells.

Recently, another approach directly targeting tumor infiltrating lymphocytes (TILs) has also started to show some clinical potential [52]. Such treatment, however, requires intra-lesional injection of IL-2/IL-15/IL-21 for successful expansion and activation of TILs in GBM patients. As compared to GBM the level of CD8^+^ cytotoxic T cells are higher in MNG therefore such treatment might lead to expansion and activation of the cytotoxic T cell pool and becomes a viable treatment approach for inoperable MNG. Further studies, however, are certainly required for the clinical approval of such therapeutic approach either in GBM or MNG, as not only CD8^+^ cytotoxic T cells and NK cells respond with clonal expansion to an IL-2/IL-15 cocktail, but the dominantly present immunosuppressive T cells as well, which might explain the variable success of such therapeutic attempts so far [53].

Admittedly, the sample size in this study is fairly small; therefore future studies will be required to determine the extent to which the immune microenvironment of meningiomas and glioblastomas contributes to the severity and clinical outcomes of CNS tumors.

## Figures and Tables

**Table 1:** Summary of patient data—tumor type, age range, diagnosis and therapy preceding surgery (N/A = not applicable; (-) = negative).

Tumor Type	N°	Code	Age Range	IDH	Diagnosis	Radio- or Other Therapy before Surgery
Meningioma	1	M1	50–60	N/A	Grade I meningioma	-
	2	M2	70–80	N/A	Grade I meningioma	-
	3	M3	40–50	N/A	Grade I meningioma	-
	4	M4	70–80	N/A	Grade I transitional meningioma	-
	5	M5	60–70	N/A	Grade I meningioma brain invasion	-
	6	M6	40–50	N/A	Grade I meningioma	-
	7	M7	40–50	N/A	Grade I meningioma	+
	8	M8	70–80	N/A	Grade I meningioma	-
Glioblastoma	1	G1	60–70	(-)	Grade IV Glioblastoma	-
	2	G2	70–80	(-)	Grade IV Glioblastoma	-
	3	G3	60–70	(-)	Grade IV Glioblastoma	+
	4	G4	40–50	(-)	Grade IV Glioblastoma	-
	5	G5	60–70	(-)	Grade IV Glioblastoma	-

**Table 2:** List of gene specific primer sequences used in qRT-PCR.

Target Genes	Accession Number	Primers Sequences
**beta-Actin**	NM_001101.5	
Forward		5′-GCGCGGCTACAGCTTCA-3′
Reverse		5′-CTTAATGTCACGCACGATTTCC-3′
**GAPDH**	NM_002046.7	
Forward		5′-ATCCCTCCAAAATCAAGTGA-3′
Reverse		5′-GGCTGTTGTCATACTTCTCA-3′
**FOXP3**	XM_006724533.2	
Forward		5′-AAGGACAGGTCAGTGGACAG-3′
Reverse		5′-CGAAGACCTTCTCACATCCG-3′
**IDO1**	NM_002164.5	
Forward		5′-CCAAGAAACTGGAACTGCCT -3′
Reverse		5′-CTGCAGTCTCCATCACGAAA-3′
**IL-10**	NM_000572.3	
Forward		5′-CCTGCCTAACATGCTTCGAG-3′
Reverse		5′-GGTCTTGGTTCTCAGCTTGG-3′
**INF- gamma**	NM_000619.2	
Forward		5′-GAATGTCCAACGCAAAGCAA-3′
Reverse		5′-ACCTCGAAACAGCATCTGAC-3′
**CD27**	>NM_001242.4	
Forward		5′-TGCAGAGCCTTGTCGTTACAG-3′
Reverse		5′-GCTCCGGTTTTCGGTAATCCT-3′
**CD163**	XM_024449278.1	
Forward		5′-GGACAGGGTTAGGGAGTCAT-3′
Reverse		5′-TAAGCTGCTGGCAAAGAACA-3′
**CTLA4**	NM_005214.5	
Forward		5′-ATGTACCCACCGCCATACTA-3′
Reverse		5′-CGAACTAACTGCTGCAAGGA-3′
**CD28**	XM_011512194.2	
Forward		5′-GCCTTGGCAGGAAACAAGAT-3′
Reverse		5′-AGTCCTTTGTGAAGGGATGC-3′
**TGF-beta**	NM_000660.6	
Forward		5′-GACATCAACGGGTTCACTACC-3′
Reverse		5′-CGTGGAGCTGAAGCAATAGTT-3′
**CD4**	NM_001195014.2	
Forward		5′-TGCACCCTCATCTTCCTATCT-3′
Reverse		5′-AGGAGAACTCCACCTGTTCC-3′
**PD-1**	NM_005018.3	
Forward		5′-CAGTTCCAAACCCTGGTGGT-3′
Reverse		5′-GGCTCCTATTGTCCCTCGTG-3′
**PD-L1**	NM_014143.4	
Forward		5′-ATGGTGGTGCCGACTACAAG-3′
Reverse		5′-GGAATTGGTGGTGGTGGTCT-3′
**CD19**	XM_006721103.3	
Forward		5′-CAGGGTCCCAGTCCTATGAG-3′
Reverse		5′-TCTGGCCCATCGGGATTAT-3′
**CD56**	NM_001242608.1	
Forward		5′-TAGTTCCCAGCTGACCATCA-3′
Reverse		5′-TGGCAGTCTGGTTCTCTACA-3′
**CD3**	NM_000733.3	
Forward		5′-ATGTCTGCTACCCCAGAGGA-3′
Reverse		5′-GTTTTGTCCCCTTTGCCTGC-3′
**CD8**	NM_001145873.1	
Forward		5′-ACCCTTTACTGCAACCAC-3′
Reverse		5′-TTGTCTCCCGATTTGACCAC-3′
**PAX5**	NM_016734.3	
Forward		5′-GTAGTCCGCCAGAGGATAGT-3′
Reverse		5′-TCCAATTACCCCAGGCTTGA-3′
**CD70**	NM_001330332.2	
Forward		5′-GGCATCTACATGGTACACATCC-3′
Reverse		5′-ACTTGACTTTGAGTCCCCAG-3′
**B7-1**	NM_005191.4	
Forward		5′-CAGGTGTTATCCACGTGACC-3′
Reverse		5′-CCTTTTGCCAGTAGATGCGA-3′
**B7-2**	NM_175862.5	
Forward		5′-CACAGCAGAAGCAGCCAAAATG-3′
Reverse		5′-CTTCAGAGGAGCAGCACCAGA-3′

**Table 3:** List of antibodies used for protein detection.

Antibody	Clone	Source	Isotype	Source	Dilution
Anti-CD4	4B12	Mouse	IgG1, kappa	Thermo Fisher Scientific	1:20
Anti-CD8	C8/114B	Mouse	IgG1, kappa	Thermo Fisher Scientific	1:50
Anti-CD3	Polyclonal	Rabbit	/	Dako	1:400
Anti-CD45	2B11 + PD7/26	Mouse	IgG1, kappa	Dako	1:400
Anti-CD19	EPR5906	Rabbit	IgG	Abcam	1:500–1:1000
Anti-CD68	PGM1	Mouse	IgG3, kappa	Dako	1:200
Anti-PD1	NAT105	Mouse	IgG1, kappa	Abcam	1:50
Anti-PDL1	22C3	Mouse	IgG1	Dako	1:50

## Data Availability

The data presented in this study are available on request from the corresponding author. The data are not publicly available due to privacy issues.

## Supplementary Materials

The following are available online at https://www.mdpi.com/1873-149X/28/1/4/s1. Figure S1: (A) mRNA expression levels of PAX5 in MNG and GBM patient samples. Data are presented as 1/dCt individually and as average ± SEM. (B,C) Immunohistochemistry staining of PAX5 and CD79a proteins in both MNG and GBM tumors, magnification ×20 and ×40, size bar 100 and 20 μm, respectively; Figure S2: Immunohistochemistry staining of CD3, CD45, CD8, CD4, PAX5 and PGM1 in normal human brain tissues (NHB). Magnification ×20, size bar 100 μm; Table S1: Quantification of infiltrating immune cells in mm^2^ tissue area. CD3+ CD4+ CD8+ and CD68+ positive cells/mm^2^ in MNG and GBM IHC stained tissues.

## Abbreviations

CNS	Central nervous system
GBM	Glioblastoma
MNG	Meningioma
Treg	Regulatory T cell
TAM	Tumor-associated macrophages
IDO1	Indoleamine 2,3-dioxygenase 1
LGG	Low-grade gliomas
TMZ	Temozolomide
NK	Natural killer
MDSSC	Myeloid-derived suppressor cells
BBB	Blood brain barrier
GAPDH	Glyceraldehyde 3-phosphate dehydrogenase
TBS	Tris Buffer Saline
BSA	Bovine serum albumin
INF-γ	Tumor interferon gamma
TGFβ	Transforming growth factor β
TNF	Tumor necrosis factor
PD-1	Programmed cell death protein 1
PD-L1	Programmed death-ligand
CTLA-4	Cytotoxic T-lymphocyte antigen 4
CD80	B7-1
CD86	B7-2
TIL	Tumor infiltrating lymphocyte
