# Supplementary material for: Is an Immunosuppressive Microenvironment a Characteristic of Both Intra- and Extraparenchymal Central Nervous Tumors?
Source: Pathophysiology. 2021 Jan 8;28(1):34–49. doi: 10.3390/pathophysiology28010004 (PMC8830452; doi:10.3390/pathophysiology28010004)
Supplement: Supplementary file 1 [file pathophysiology-28-00004-s001.pdf]

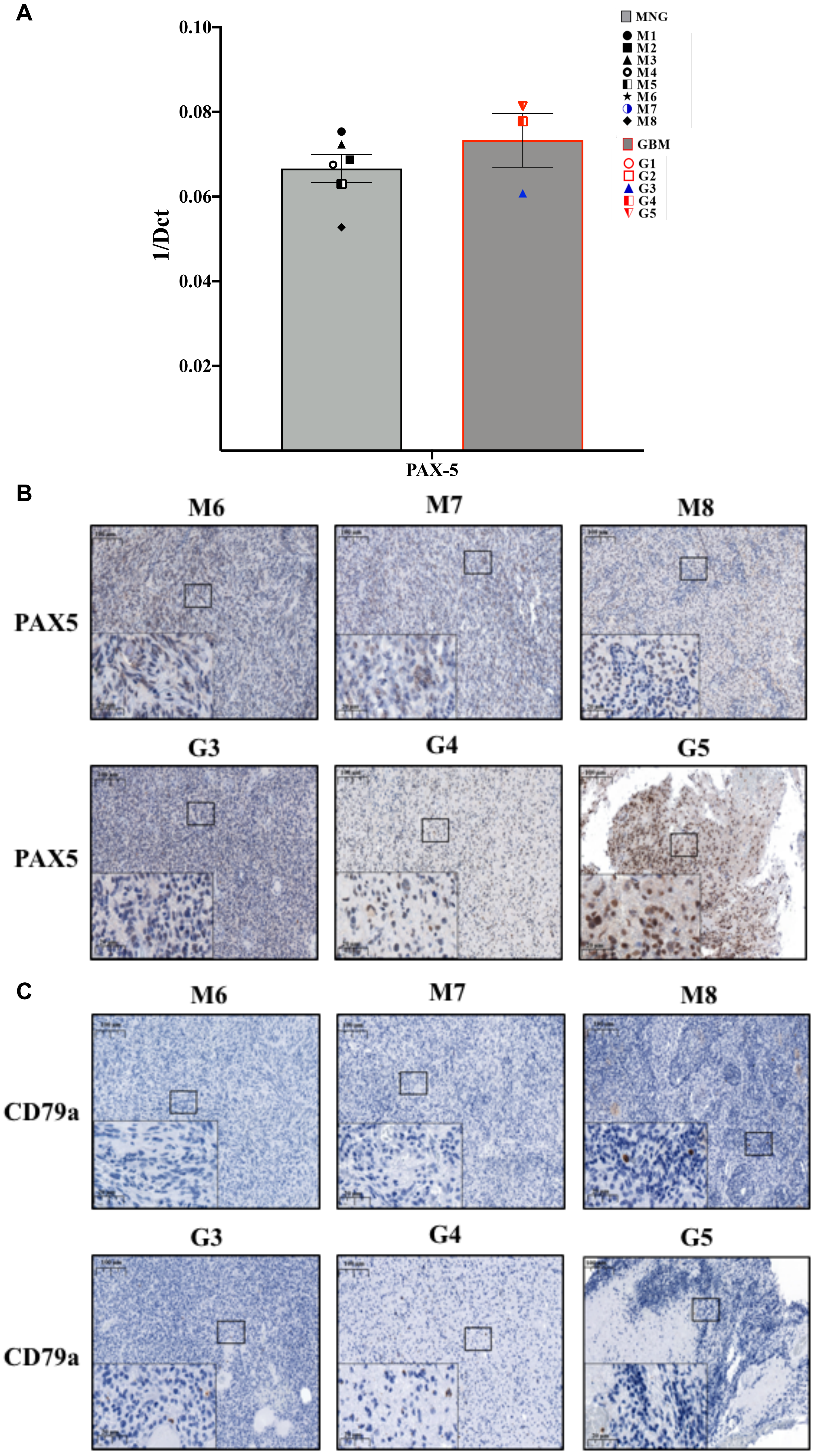

Supplementary Figure S1. A) mRNA expression levels of PAX5 in MNG and GBM patient samples. Data are presented as 1/dCt individually and as average±SEM. **B, C)** Immunohistochemistry staining of PAX5 and CD79a proteins in both MNG and GBM tumors, magnification x20 and x40, size bar 100 and 20 µm respectively.

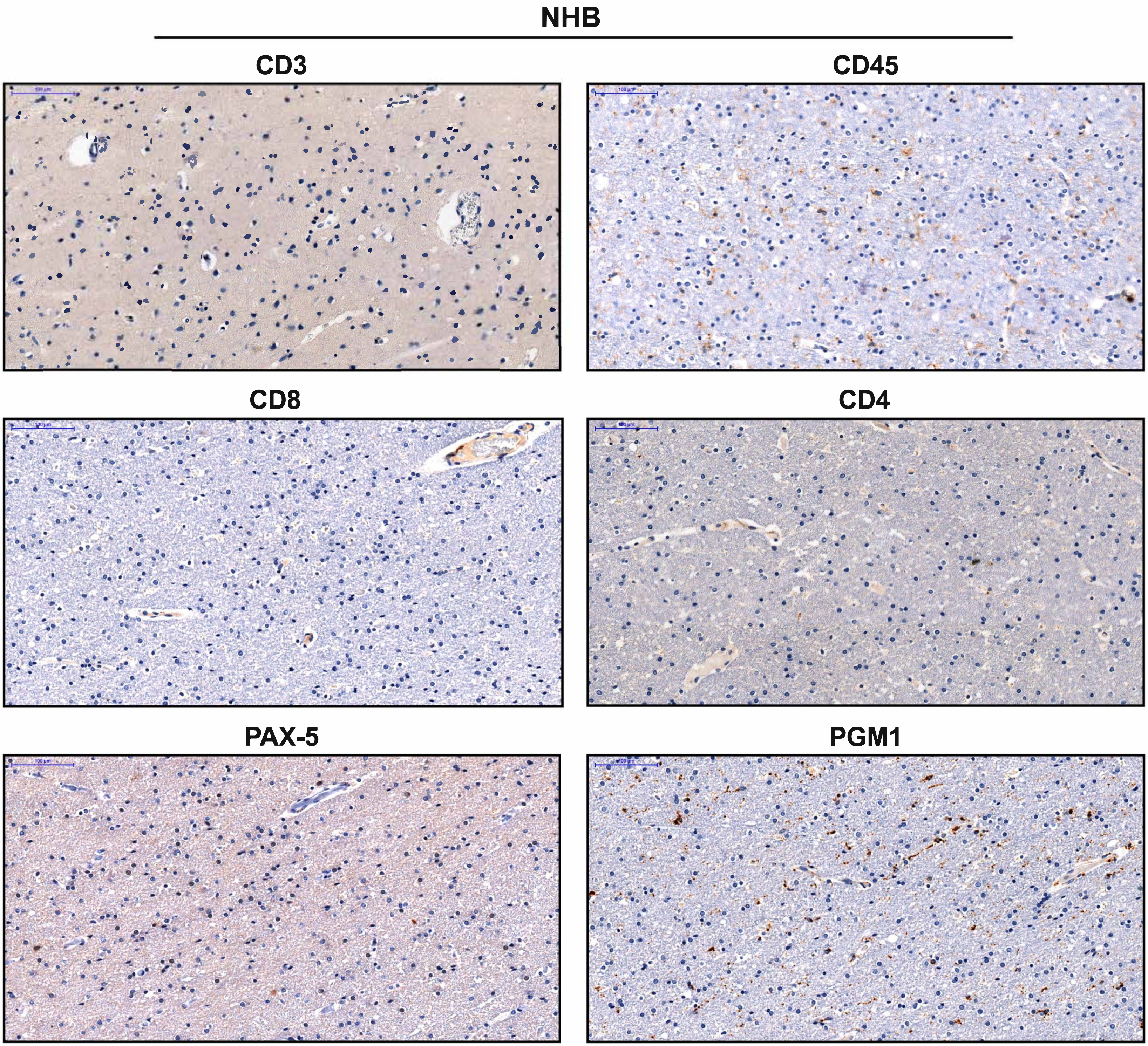

Supplementary Figure S2. Immunohistochemistry staining of CD3, CD45, CD8, CD4, PAX-5 and PGM1in normal human brain (NHB). Magnification x20, size bar 100 µm.

				per mm2							per 0.08 mm2		
**Code**	**Freeze**	**Localization**	**Comment**	**CD3**	**CD4**	**CD8**	**CD4/CD8**	**PD1**	**PD-L1**	**CD68**	**CD68**	**PAX5**	**CD79a**
**M6**	Yes	Left posterior fossa	Focally lymphocytes	210	12	40	0.3	12	0%**	High	100	0	0
**M7**	Yes	Right frontal and orbital	Tumor cells focally CD4+	150	18	25	0.72	9	<1%	High	100	0*	0
**M8**	Yes	Over left sphenoidal bone	Tumor cells focally CD4+	54	9	9	1	<1	<1%*	Low	40	0*	2
**G3**	Yes	Left temporal	Focally lymphocytes	30	6	6	1	<1	<1%	Moderate	40	30	1
**G4**	Yes	Left occipital		9	3	2	1.5	<1	0%	Moderate	40	0*	2
**G5**	Yes	Right centrum semiovale		60	27	7	3.857143	18		Moderate	40	0**	1
**G6**	Yes	Left temporal		120-600	180	50	3.6	90	-	High	100		

M: Meningioma

G: Glioblastoma

** moderate to strong staining in tumor cells

* weak staining in tumor cells

** Lymphocytes show strong positivity in one focus

* Macrophages are positive

Supplementary Table S1. Quantification of infiltrating immune cells in mm^2 tissue area. CD3$^+$ CD4$^+$ CD8$^+$ and CD68$^+$ positive cells/mm2 in MNG and GBM IHC stained tissues.
